# *CLOCK *is suggested to associate with comorbid alcohol use and depressive disorders

**DOI:** 10.1186/1740-3391-8-1

**Published:** 2010-01-21

**Authors:** Louise K Sjöholm, Leena Kovanen, Sirkku T Saarikoski, Martin Schalling, Catharina Lavebratt, Timo Partonen

**Affiliations:** 1 Department of Molecular Medicine and Surgery, Karolinska Institutet, Neurogenetics Unit CMM L8:00 Karolinska University Hospital, 171 76 Stockholm, Sweden; 2 Department of Mental Health and Substance Abuse Services, National Institute for Health and Welfare, PO Box 30, 00271 Helsinki, Finland; 3 Department of Alcohol, Drugs and Addiction, National Institute for Health and Welfare, PO Box 30, 00271 Helsinki, Finland

## Abstract

**Background:**

Depression and alcohol abuse or dependence (AUD) co-occur in the general population more frequently than expected by chance. Alcohol use influences the circadian rhythms generated by the central pacemaker in the suprachiasmatic nucleus, and circadian rhythm alterations in turn are common in depressive disorders as well as among persons addicted to alcohol.

**Methods:**

32 SNPs in 19 circadian clockwork related genes were analyzed using DNA from 76 individuals with comorbid depression and AUD, 446 individuals with AUD and 517 healthy controls with no psychiatric diagnosis. The individuals participated in a nationwide health examination study, representative of the general population aged 30 and over in Finland.

**Results:**

The *CLOCK *haplotype TTGC formed by SNPs rs3805151, rs2412648, rs11240 and rs2412646, was associated with increased risk for comorbidity (OR = 1.65, 95% CI = 1.14-2.28, P = 0.0077). The SNPs of importance for this suggestive association were rs2412646 and rs11240 indicating location of the functional variation in the block downstream rs2412648. There was no indication for association between *CLOCK *and AUD.

**Conclusion:**

Our findings suggest an association between the *CLOCK *gene and the comorbid condition of alcohol use and depressive disorders. Together with previous reports it indicates that the *CLOCK *variations we found here may be a vulnerability factor to depression given the exposure to alcohol in individuals having AUD.

## Background

Depression, alcohol abuse or dependence (AUD), as well as other affective disorders and substance use disorders (SUD), co-occur in the general population more frequently than expected by chance [1,2]. Approximately 80% of individuals with AUD report symptoms of depression and 25-40% of the people suffering from depression also report drinking problems [3]. The comorbidity of depression and AUD complicates the treatment and can alter the prognosis [3,4]. Furthermore, both depression and AUD increase the risk of suicide. Hence, having both depression and AUD is more severe than having just one of the disorders and it often leads to greater impairment [5,6].

A number of hypotheses have been proposed to explain the comorbidity between depression and AUD and answer to whether we are dealing with one or two independent and overlapping disorders. The comorbidity could be due to shared risk factors or highly correlated risk factors [1]. Also, some symptoms of AUD overlap with some common symptoms in depression, such as sadness and sleep disturbances [3]. It has also been discussed whether the co-occurrence could be the result of one of the disorders increasing the risk for or even aggravating the other disorder [1,3]. Alcohol dependent individuals are possible at higher risk of developing depression, as a consequence of the associated interpersonal and social problems often caused by alcohol dependence [1]. On the contrary, the substance induced mood disorder theory advocates that depressed persons are more vulnerable to develop an addiction/abuse. Related to this latter assumption is the self medication theory, where the depressed individual tries to self-medicate with alcohol [1-3].

Depression and AUD are both complex disorders meaning that both genetic and environmental risk factors have an influential role, with the interplay between genes of modest effect with several environmental risk factors contributing to disease susceptibility. Results from several studies indicate that both environmental and genetic risk factors partly overlap between depression and AUD suggesting a common etiology [7,8]. An epidemiological study by Prescott and colleagues on depression and alcoholism conclude that the causes overlap between the disorders, though without having the same origin and they estimated that the shared overlap of genetic and environmental factors influencing depression and AUD was only 9-14% [4]. Nevertheless, several genes have been proposed to be involved in the etiology of both depression and AUD, exemplified by brain-derived neurotrophic factor *(BDNF*), neuropeptide Y (*NPY*), dopamine receptor D2 (*DRD2*), catechol-O-methyltransferase (*COMT*), monoamine oxidase A (*MAOA*), period homolog 2 (*PER2*) and several subtypes of the serotonin receptor [8-12]. Only few studies have investigated the genetic component of the co-occurrence between depression and AUD. McEachin et al. investigated the comorbidity between depression and AUD *in silico*, by modeling gene-by-environment interactions using bioinformatics and identified tumor necrosis factor (*TNF*) and methylenetetrahydrofolate reductase (*MTHFR*) as candidate genes. TNF is involved in the pathway activating MTHFR expression and excessive alcohol intake leads to reduced TNF signaling. MTHFR is a key component of the folate metabolism and previously folate levels have been associated to both depression and AUD [7]. In addition, linkage to chromosome 1p13-35 locus and alcoholism or depression has been found, the region containing several genes includes two genes coding for potassium channel-related proteins [13].

Alcohol is known to influence and alter the circadian rhythm and it may even act on the central pacemaker located in the suprachiasmatic nucleus (SCN) [14]. Studies also show that alcohol preference and sensitivity vary along with the circadian oscillation [15,16]. Studies show that rats and other rodents have a preference for alcohol during their active phase (dark-phase) [15]. Drug-induced changes of gene expression have been reported for several clock genes and the CLOCK:ARNTL transcription activity was increased in *in vivo *experiments when stimulating the dopamine D2 receptor [16]. The Period (*Per*) genes in rats have an decreased circadian expression pattern in SCN and various other brain areas after alcohol intake [17]. Spanagel and colleagues found that a haplotype in the *PER2 *gene associated with high (>300 g/day) versus low (<300 g/day) alcohol intake, though it was not associated with alcohol dependence [18]. Also, both non-seasonal and seasonal unipolar depressive and bipolar disorders and certain sleep disorders are associated with an abnormal circadian rhythm and display symptoms like disturbed sleep-wake cycle and appetite, as well as abnormal physical functions including changes in temperature and various hormonal levels [19-21].

Our aim here was to investigate whether certain genetic variations in the circadian clock system are associated with comorbidity between depression and AUD.

## Methods

### Material

The study groups were selected from the Health2000 study which is a population based Finnish nationwide health interview and examination survey (for more information, see http://www.terveys2000.fi/indexe.html or http://www.kela.fi/in/internet/liite.nsf/%28WWWAllDocsById%29/947B8325F4EF9801C225744A0029D9BC/$file/tutkimuksia86.pdf. The individuals with both a depression diagnosis and an alcohol use diagnosis (AUD), n = 76, were selected, as well as the 446 individuals with AUD only (without other mental disorder) and 517 sex and age-matched healthy controls with no psychiatric symptom (Table 1). The depression and AUD diagnoses were based on the Composite International Diagnostic Interview (M-CIDI) and diagnoses were set according to the DSM-IV criteria (codes: 296.2× or 296.3× major depressive disorder, 300.4 dysthymia, 305.00 alcohol abuse, 303.90 alcohol dependence). The individuals with depression and AUD comorbidity (cases) were compared to healthy individuals (controls) referred to as Sample set 1. Two additional sample sets were used to investigate possible findings in Sample set 1. All the sample sets are displayed in Table 2.

**Table 1:** Descriptive statistics of the study group.

Diagnosis			Number (n)	Mean age ± **SEM**	Females %
Depression or Dysthymia + Alcohol dependence or abuse diagnosis (AUD)			76	46.6 ± 1.220	43.3
Individuals with an alcohol dependence or abuse diagnosis (AUD)			446	47.1 ± 0.550	15.1
Individuals without an alcohol or dependence diagnosis (AUD)			517	46.2 ± 0.494	19.5
Total			1039		

**Table 2:** The Sample sets used.

Sample sets	Cases number n	Controls number n
1. Depression or Dysthymia + AUD diagnosis vs individuals without psychiatric symptoms	76	517
2. Individuals with an AUD diagnosis vs individuals without psychiatric symptoms	446	517
3. Depression or Dysthymia + AUD diagnosis vs individuals with and without an AUD diagnosis	76	963

The SNPs found nominally associated (P < 0.05) in Sample set 1 were investigated in Sample sets 2 and 3.

Table 3 describes the comorbid cases and healthy controls based on the modified 6-item Global Seasonality Score (GSS) [22], the 21-item modified Beck Depression Inventory (BDI) [22], the 12-item General Health Questionnaire (GHQ) [22], the 16-item Maslach Burn Out Inventory - General Survey (MBI) [23] and the length of sleep per day. The GSS assesses seasonal changes in mood and behavior. In the modified GSS questionnaire each of the six items was scored from 0 to 3 (none, slight, moderate or marked change), with higher scores indicating greater seasonal changes. The modified questionnaire was good in representing the adult Finnish population, the scores of 0 to 7 assigned as low and those of 8 to 18 as high [24]. Modification was made to the 21-item BDI giving a sum score ranging from 0 to 55. The modified BDI was validated in the Finnish population where the scores of 0 to 9 assigned as low and 10 to 55 as high degree of depressive symptoms. The GHQ scale evaluates whether the individual complains about a recent symptom or behavior. GHQ is a valid screening tool and a measure of psychological distress at population level, especially concerning anxiety and depression. According to the analysis of data derived from the Health 2000 Health Examination Survey, the scores of 0 to 4 assigned as low and those of 5 to 36 as high mental ill-being. The MBI score for burnout was weight calculated as 0.4 × exhaustion + 0.3 × cynicism + 0.3 × diminished professional efficacy. A score of >1.49 indicates burnout and corresponds to symptoms on a monthly basis or more frequently. In this study, the GSS, BDI, GHQ and MBI scores were significantly higher for cases than controls (Sample set 1) (P < 0.0001).

**Table 3:** Descriptive statistics of the continuous variables for Sample set 1.

Variable name	Score range	Group	Total n	Min-Max	Median
6-item Global Seasonality Score* (GSS)	0-18	Cases	76	0-13	6.00
		Controls	517	0-18	4.00
21-item Beck Depression Inventory* (BDI)	0-55	Cases	76	0-44	18.50
		Controls	517	0-24	3.00
12-item General Health Questionnaire* (GHQ)	0-36	Cases	76	0-12	7.00
		Controls	517	0-12	0.00
Maslach Burnout Inventory-General* (MBI)	0-16	Cases	76	0.26-4.73	2.18
		Controls	517	0.00-3.88	0.79
Length of night sleep (h)	4-12	Cases	76	4-12	6.00
		Controls	517	4-11	7.00

* GSS, BDI, GHQ and MBI scores were higher for cases than controls in Sample set 1 (P < 0.0001).

### Single-nucleotide polymorphism (SNP) selection and genotyping

In total, 39 SNPs in 20 circadian clockwork related genes were selected. Candidate SNPs, with a possible functionality (e.g. amino acid changes or published data on functional alterations) and/or prior published associations to substance use or mental health disorders, in circadian clock genes but also genes upstream or downstream in circadian pathways were selected. In addition, tagSNPs from HapMap were selected, to increase the variation coverage within core clock genes. Genomic DNA was isolated from whole blood according to standard procedures. SNPs were genotyped with a fluorogenic 5' nuclease assay method (TaqMan™) with both pre-designed and custom made primer-probe kits (TaqMan^® ^Pre-Designed SNP Genotyping Assays, Applied Biosystems, Foster City, CA, USA) using Applied Biosystems 7300 Real Time PCR System (Applied Biosystems) according to manufacturers' instructions. Custom made assays were made for Adenosine deaminase (*ADA*) 22G>A (Asp8Asn), farnesyl-diphosphate farnesyltransferase 1 (*FDFT1*) rs11549147 and *PER2 *10870 (Additional file 1, Table S1). Of the 39 SNPs, rs35878285 in *ARNTL2*, S662G in *PER2 *and rs2863712 in *CRY2 *and rs2230783 in *NCOA3 *were non-polymorphic. The rs934945 in *PER2*, and rs6486120 and rs1982350 in *ARNTL *were also excluded as the controls were not (P < 0.05) in Hardy-Weinberg equilibrium (HWE). Finally, 32 SNPs in 19 genes were further analyzed (Table 4). All laboratory procedures were carried out blind to case/control status. Five percent of the samples were regenotyped and showed no error.

**Table 4:** The 19 circadian clockwork related genes and the 32 SNPs analyzed.

Gene	Gene name	Location	ID (rs#) of SNPs genotyped
*ARNTL*	*Aryl hydrocarbon receptor nuclear translocator-like*	11p15.2	(rs2290035, rs3816360, rs2278749)

*ARNTL2*	*Aryl hydrocarbon receptor nuclear translocator-like 2*	12p12.2-p11.2	(rs4964057, rs2306074, rs7958822, rs1037921)

*CLOCK*	*Clock homolog (mouse)*	4q12	(rs3805151, rs2412648, rs11240, rs2412646)

*NPAS2*	*Neuronal PAS domain protein 2*	2q11.2	(rs11541353, rs2305160)

*PER2*	*Period homolog 2 (Drosophila)*	2q37.3	(Spanagel/10870, rs2304672)

*TIMELESS*	*Timeless homolog (Drosophila)*	12q13.2	(rs2291739, rs2291738)

*ACADS*	*Acyl-Coenzyme Adehydrogenase*, *C-2 to C-3 short chain*	12q22-qter	(rs1799958/rs17848088)

*ADA*	*Adenosine deaminase*	20q12-q13.11	(Asp8Asn)

*ADCYAP1*	*Adenylate cyclase activating polypeptide 1 (pituitary)*	18p11.32	(rs2856966)

*DRD2*	*Dopamine receptor D2*	11q23.1	(rs6277)

*ANKK1*	*Ankyrin repeat and kinase domain containing 1*	11q23.1	(rs1800497)

*FDFT1*	*Farnesyl-diphosphate farnesyltransferase 1*	8p23.1-p22	(rs11549147)

*GLO1*	*Glyoxalase I*	6p21.3-p21.1	(rs2736654)

*OPN4*	*LIM domain binding 3;opsin 4 (melanopsin)*	10q23.2	(rs1079610)

*NCOA3*	*Nuclear receptor coactivator 3*	20q13.12	(rs6094752, rs2230782)

*NPY*	*Neuropeptide Y*	7p15.1	(rs16139)

*PLCB4*	*Phospholipase C, beta 4*	20p12	(rs6077510)

*VIP*	*vasoactive intestinal peptide*	6q25.2	(rs3823082, rs688136)

*VIPR2*	*Vasoactive intestinal peptide receptor 2*	7q36.3	(rsS885863)

Symbol approved by the HUGO Gene Nomenclature Committee (HGNC) database http://www.genenames.org/, the location and rs# were taken from the NCBI Entrez Gene and dbSNP BUILD 129 database respectively http://www.ncbi.nlm.nih.gov/. TagSNPs were selected using HapMap (The International HapMap Consortium, 2005). Note: Historically *DRD2 Taq1A *(rs1800497) has been assigned to *DRD2 *whereas more recent data have indicated that the SNP is actually located within the coding region of *ANKK1*.

### Statistical analyses

HWE for all SNPs were calculated for the control group. Allele and genotype frequency differences between cases and controls (Sample set 1) were tested using logistic regression controlling for gender, due to over-representation of females in the case group (Table 1) applying the PLINK program http://pngu.mgh.harvard.edu/purcell/plink/, version 1.05 [25] and the R software http://www.r-project.org/, version 2.10.0, package stats [26], respectively. To obtain empirical significance, permutation tests with 10,000 permutations were calculated. SNPs which showed nominal association (P < 0.05, allelic or genotypic) were tested for association also in Sample sets 2 and 3 (Table 2).

The linkage disequilibrium (LD) measure D' was calculated among the controls and blocks were constructed using the Haploview program, version 3.2 [27] using the block parameters [28] and the D' confidence interval algorithm in the Haploview program. Test for haplotype frequency difference between cases and controls in Sample set 1 was performed for the haplotype blocks harboring nominally associated SNPs (P < 0.05), being one block in *CLOCK*. Nominal haplotype association in Sample set 1 was then also analyzed in Sample sets 2 and 3. The PLINK program was used to perform the calculations and gender was controlled for.

With the number of tests being performed in this study an ordinary Bonferroni correction seemed conservative. Also it does not take into account the selection process being used in this study, i.e. choosing genes and SNPs based on biological relevance. Nor does the Bonferroni correction consider the LD between the SNPs [29]. Therefore, threshold for significance was calculated using a Bonferroni correction considering the partial LD between SNPs. The nominal significance level was divided by the number of SNP-groups (24) defined by D'>0.80 among the controls [30,31]. The difference in allele, genotype or haplotype frequency for the three Sample sets was regarded significant if P < 0.0021 (0.05 divided by 24).

Power to detect allele frequency difference for Sample set 1 was ≥ 0.8 for an OR ≥ 2.0 at allele frequency ≥ 0.3, or for an OR ≥ 2.2 at allele frequency ≥ 0.2. For Sample set 2, the power to detect allele frequency difference was ≥ 0.8 for an OR of ≥ 1.5 at allele frequency ≥ 0.3, or for an OR of ≥ 1.6 at allele frequency ≥ 0.2.

The mean values for the continuous variables were compared between cases and controls (Sample set 1) using the Mann-Whitney U test, since the variables were not normally distributed in the control group, in PASW version 18.0.0 [32]. Significant difference was set to P < 0.05/4 (four variable groups with r < 0.6). Where difference between cases and controls were found, association between the variable and the nominally associated SNPs were investigated with univariate linear regression with SNP as predictor using PLINK. Gender was not included since it did not contribute to the genetic additive model.

## Results

Allele and genotype frequencies of SNPs in circadian genes (Table 4) were tested for association with depression and AUD comorbidity as compared to healthy controls (Sample set 1, Table 2). For SNPs with nominal allele or genotype association (P < 0.05), genetic association analyses with AUD versus healthy controls (Sample set 2) and comorbidity versus AUD and controls (Sample set 3) were performed to determine the nature of the association to the comorbidity. The results are presented in Table 5.

**Table 5:** SNP allele and genotype frequency association analysis for the three Sample sets.

Gene	Function	SNP	Alleles	Sample set	MAF A/U	OR (95% CI)*	P-values allele		P-values genotype		
							*	Empirical	Cochran- Armitage trend*	Dominant model*	Recessive model*
*CLOCK*	Intron	rs11240	G/C	1.	0.44/0.33	1.65 (1.14-2.38)	0.0077	0.0072	0.0077	0.055	0.013
				2.	0.33/0.33	1.02 (0.85-1.24)	Ns	Ns	Ns	Ns	Ns
				3.	0.44/0.33	1.59 (1.13-2.26)	0.0084	0.0068	0.0082	0.048	0.016
*ARNTL2*	Intron	rs2306074	C/T	1.	0.30/0.35	0.77 (0.53-1.12)	Ns	Ns	Ns	0.043	Ns
				2.	0.33/0.35	0.90 (0.74-1.09)	Ns	Ns	Ns	Ns	Ns
				3.	0.30/0.34	0.80 (0.55-1.15)	Ns	Ns	Ns	0.056	Ns
*ACADS*	Mis-sense mutation	rs1799958	A/G	1.	0.34/0.26	1.47 (1.01-2.15)	0.045	0.044	0.045	0.097	Ns
				2.	0.28/0.26	1.11 (0.91-1.37)	Ns	Ns	Ns	Ns	Ns
				3.	0.34/0.27	1.44 (1.01-2.07)	0.046	0.040	0.046	0.097	Ns

SNPs which showed nominal association (allelic or genotypic) P < 0.05 for Sample set 1 are displayed as are the p-values P < 0.1. Ns = non significant. Analysis in Sample set 2 and 3 were then performed for these SNPs. Alleles: the minor allele first. Odds ratio (OR): the proportion of minor versus major allele in the affected (A) divided by the proportion of minor versus major allele in the non-affected (U) individuals. Empirical P is the point-wise P-value after 10,000 permutations.* gender was used as covariate.

In the *CLOCK *gene the minor allele G of rs11240 was suggestively associated with depression and AUD comorbidity and showed an OR of 1.65 (P = 0.0077). This was further strengthened by the results from the recessive model (GG vs GC and CC, P = 0.013) and trend test (P = 0.0077). Rs11240 showed no associations to AUD in Sample set 2. Accordingly, in Sample set 3 the allele G was suggestively associated with risk for depression (OR = 1.59, P = 0.0084) and the Cochran-Armitage test suggested a trend (P = 0.0082).

One LD block was formed in *CLOCK *(rs3805151, rs2412648, rs11240, rs2412646), spanning a 18-kb region, using Sample set 1 data. The haplotype TTGC, including the rs11240 risk allele G, suggestively conferred a risk for comorbidity (OR = 1.65, P = 0.0077) (Table 6). This TTGC haplotype was suggestively associated also with an increased risk for depression in Sample set 3 (OR = 1.50, P = 0.0084). Three additional haplotypes were formed, however none of then nominally associated, TTCT, CGCC and CTCC with overall frequencies of 26%, 36% and 3.8% respectively. To elucidate TT alleles role in the two haplotypes, analysis was performed with only the last two SNPs in the haplotype (rs11240 and rs2412646). The p-values for the risk haplotypes did not change between the TTGC and the GC haplotypes in Sample set 1 (Table 6) indicating location of the functional variation downstream of rs2412648.

**Table 6:** Haplotype association analysis of *CLOCK*.

SNP block		Haplotype	Sample set	Overall frequency	OR (95% CI)	P-value
rs3805151-rs2412648-rs11240-rs2412646		TTGC	1.	0.34	1.65 (1.14-2.28)	0.0077
			2.	0.33	Ns	Ns
			3.	0.34	1.50 (1.14-2.27)	0.0084
rs11240-rs2412646		GC	1.	0.34	1.65 (1.15-2.29)	0.0077
			2.	0.28	Ns	Ns
			3.	0.34	1.50 (1.14-2.27)	0.0084

Ns = non significant (P > 0.1). Odds ratio (OR): the ratio specific haplotype versus all other haplotypes among the cases, relative to the ratio specific haplotype versus all other haplotypes among the controls. Gender was used as covariate.

The rs2306074 in *ARNTL2 *showed a border-line nominal Cochran-Armitage trend in Sample set 1 (P = 0.043), and no indication for association in Sample sets 2 and 3. In *ACADS*, the A allele of rs1799958 showed a border-line nominal allelic association of increased risk for comorbidity (OR = 1.47, P = 0.045) and a trend test supported the finding (P = 0.045). Likewise, in Sample set 3 a very modest nominal allelic association was found for rs1799958 (P = 0.046). No haplotype could be constructed for *ARNTL2 *or *ACADS *that included the SNPs nominally associated with comorbidity.

To test for a quantitative effect of the *CLOCK *rs11240, *ACADS *rs1799958 and *ARNTL2 *rs2306074 on AUD depression comorbidity, these variations were tested for association to GSS, BDI, GHQ and MBI among the comorbid cases only. The A allele of rs1799958 in *ACADS *was nominally associated with higher GHQ score among cases (P = 0.031).

## Discussion

Our results herein suggest that the circadian gene *CLOCK *is associated with comorbid depression and AUD, but not with AUD only. The haplotype TTGC formed by SNPs rs3805151, rs2412648, rs11240, rs2412646 was suggestively associated with increased risk for the comorbidity, with the odds ratio of 1.65. The SNPs of importance for this suggestive association were rs11240 and rs2412646, indicating location of the functional variation downstream of rs2412648. No indication of association with *CLOCK *was found when comparing AUD with healthy controls. Accordingly, the suggestive association to *CLOCK *was seen when comparing comorbid cases with combined group of healthy controls and persons diagnosed with AUD. This *CLOCK *variation may be a vulnerability factor for depression given the alcohol exposure in AUD but not considerably increasing the risk for depression without AUD. This view is supported by the findings from other studies of the Finnish general population through the Health 2000 Study. They could not detect any *CLOCK *association with major depressive disorder or dysthymia [33] or anxiety disorders (Sipilä et al., submitted 2009), each using a disorder focused set of samples inclusive of all the cases. These studies analyzed rs10462028 and rs1801260 [33], and rs3749474 and rs1801260 (Sipilä et al., submitted 2009) that are in high LD with rs11240 analyzed in the current study (according to HapMap public release) [34]. Neither could we detect any indication that *CLOCK *variation was associated with AUD only. In agreement, the shared overlap of genetic and environmental factors influencing depression and AUD was estimated to only 9-14% out of which 50-60% was attributed to shared genetic factors. Also, Prescott and colleagues found no support that comorbidity arises from depression causing alcoholism or alcoholism causing depression using structural modeling in twins [4].

The *CLOCK *gene is one of the most central genes in the circadian system and has been studied in a wide range of areas due to its crucial role in creating and maintaining the body's internal rhythm. CLOCK protein exhibits a regulatory role as transcription factor over other circadian genes, like the *CRY*s and *PERs*, together with ARNTL or ARNTL2 protein. The CRY and PER complexes exhibit a regulatory role as repressors and inhibit the transcription of *CLOCK *and *ARNTL *and thereby themselves, when reaching a critical concentration. This transcription-translation feedback loop takes approximately 24 hours [21,35].

Individuals with depression or AUD often have circadian misalignment and many physiological phenomena such as the sleep-wake cycle and hormonal profiles are disrupted [36]. Sleep disturbances are also pronounced symptoms of a wide range of circadian rhythm disorders such as familial advanced sleep-phase syndrome (FASPS), delayed sleep phase syndrome (DSPS), as well as other psychiatric disorders like seasonal and non-seasonal mood disorders like, bipolar, schizophrenia as well as in drug addictions [37,38]. Furthermore, sleep deprivation and light therapy have an antidepressant effect synchronizing the sleep-wake cycle with the circadian rhythms, indicating the important role that the circadian system plays in many psychiatric disorders [19,39].

Individuals addicted to alcohol show circadian alterations, for example sleep disturbances [36]. As previously mentioned, alcohol has the ability to induce clock gene expression in different brain areas [18]. Ruby et al. showed evidence that ethanol significantly affects photic and non-photic phase-resetting responses in hamsters, critical for circadian regulation, by blocking the phase-resetting action of glutamate and increase the non-photic phase-resetting action of serotonin. This signal inhibition from ethanol was manifested through direct action in the core clock in SCN [40]. The preference and sensitivity to alcohol also seems to vary with the time of day [15].

Clinical effect of variations in *CLOCK *has been quite extensively studied. The SNP rs1801260 in *CLOCK *was shown to influence diurnal preference in healthy individuals, where C allele carriers had a higher evening preference. The same allele was also associated with the delay in sleep phase and insomnia in major depression and bipolar affective disorder patients [41,42]. Later, Serretti and colleagues also showed that the C/C genotype of rs1801260 was associate with the severity of insomnia in depressed and bipolar patients during SSRIs treatment [43]. SSRIs have earlier been reported to have circadian properties, inducing phase shift in neuronal firing in the SCN in rats [44]. The SNPs rs1801260 and rs11240 analyzed in the current study are probably reflecting association to the same functional polymorphism, as they are in LD with each other and display the same minor allele frequency (according to HapMap public release and NCBI Entrez dbSNP) [34,45]. Alcohol and other drugs of abuse modulate the dopamine neurotransmission, and McClung and colleagues showed that the *Clock *gene seems to increase the excitability of dopamine neurons and also the cocaine reward in mice with a dominant negative CLOCK protein that cannot activate transcription [46]. These *Clock*-mutant mice also display alcohol preference [47]. The *CLOCK *gene has also been proposed to be involved in the metabolic syndrome, which involves obesity and increased the risk for diabetes and heart disease [48].

Weak suggestive association to comorbidity was found for rs2306074 in *ARNTL2 *and rs1799958 in *ACADS*. A family study by Shi et al. found weak association between rapid cycling and a diurnal mood, with worse symptoms in the afternoon or evening, in bipolar subjects and SNPs in *ARNTL2 *[49]. There is also some association of *ARNTL2 *with social phobia (Sipilä et al., submitted 2009). Interestingly, there is a functional partnership between ARNTL2 and PER2 [50] that might bridge social phobia and alcohol use [51] to end in high alcohol intake. Support for involvement of *ARNTL2 *in seasonal affective disorder (SAD), a subtype of mood disorder that is closely related to AUD [52], has been reported by our group, where a SNP association was seen in both Swedish and Finnish materials (Sjöholm et al., submitted 2009). In addition, *ARNTL *has been reported by our group to show associations with depression in a Swedish population-based and case-control material [11,53]. Moreover, *NPAS2 *is indicated to associate with SAD [54,55] and *NPAS2 *and *ARNTL *or *ARNTL2 *heterodimerize and possess a transcriptional modulation function as the CLOCK/ARNTL complex [35].

*ACADS*, acyl-Coenzyme A dehydrogenase, C-2 to C-3 short chain, is an enzyme participating in the fatty acid β-oxidation and has not previously been reported to associate with mood disorder or AUD. Deficiency in ACADS leads to changes in theta oscillations during rapid eye movement (REM) sleep in mice [56], and in the majority of depressed patients disturbed sleep architecture is characterized by abnormal timing and distribution of REM and non-REM sleep stages give feedback to the SCN [57]. Furthermore, there are abnormal long-range temporal correlations in theta oscillations during wakefulness [58] and profound REM sleep abnormalities in patients with non-seasonal depressive disorder that have melancholic depressive symptoms [14,39]. On the other hand, non-REM sleep abnormalities, such as abnormal cross-correlations between facial temperatures and delta and theta frequencies, are found in patients with SAD that have atypical depressive symptoms [59]. The rs1799958 SNP (G>A) in *ACADS *results in the conversion of glycine to serine and associates with the short chain acyl-CoA dehydrogenase deficiency [60] that is characterized by lipid storage myopathy and muscle weakness.

An advantage of this study is that the individuals are derived from a big Finnish population based study of an ethnically homogenous population that is nationwide and representative of the general population aged 30 or over. We were able to investigate whether our results found with the comorbid versus control (without psychiatric symptoms), Sample set 1, reflected genetic vulnerability to AUD. A limitation in our study is the small size of the comorbid sample and the lack of a group of patients having depressive disorder only. For now, replication of the findings in independent study samples is needed as the most practical way to increase the probability of a true association.

## Conclusion

The comorbid condition of alcohol use and depressive disorders in the Finnish population was associated with *CLOCK *genetic variations and there was no indication for *CLOCK *gene association with AUD only. This finding together with previous reports indicates that the *CLOCK *variations we found here may be a vulnerability factor for depression given the exposure to alcohol in individuals having AUD.

## Competing interests

The authors declare that they have no competing interests.

## Authors' contributions

All the authors designed the study and the analysis. LK performed the genotyping. LS performed the statistical analysis. SS, MS, CL and TP as seniors guided the work. LS wrote the first draft of the manuscript, and the remaining authors reviewed the manuscript. Thus, all the authors contributed to and have approved the final manuscript.

## Supplementary Material

Additional file 1 **Table S1, list of primer sequences**. The primer and reporter sequences for the Custom TaqMan^® ^SNP Genotyping Assay. Click here for file
