# Supplementary material for: CLOCK is suggested to associate with comorbid alcohol use and depressive disorders
Source: J Circadian Rhythms. 2010 Jan 21;8:1. doi: 10.1186/1740-3391-8-1 (PMC2854106; doi:10.1186/1740-3391-8-1)
Supplement: Additional file 1 — Table S1, list of primer sequences. The primer and reporter sequences for the Custom TaqMan® SNP Genotyping Assay. [file 1740-3391-8-1-S1.DOC]

Table S1. The primer and reporter sequences for the Custom TaqMan® SNP Genotyping Assays.

|  | ***FDFT1* rs11549147** | ***PER2* 10870** | ***ADA* 22G>A (Asp8Asn)** |
| --- | --- | --- | --- |
| **Primer1** | GCTCAGCAGCAGCCTGAA | AAGCCGACTTTGCCTGAGT | CGCACGAGGGCACCAT |
| **Primer2** | CGAAACTGCGACTGGTCTGATT | ACAAGGAGCCGGGTTCTG | TGGGCCCCGCTAAGC |
| **Reporter1** | CTTGCTACAAGTATCTC | CTTGGGCATTTTCAT | ACTTTGGGCTTGTCGAA |
| **Reporter2** | TTGCTACAGGTATCTC | TTGGGCGTTTTCAT | ACTTTGGGCTTGTTGAA |
